# Annotations

**Published:** 1889-06-01

**Authors:** 


### Annotations.

According to Mr. Timothy Holmes, there was an outbreak of contagious ophthalmia in the Hanwell Schools so long ago as 1862-65 ("Holmes's Surgery," p. 663). It is now 1889, and ophthalmia in the Hanwell Schools is still exercising the minds of the local managers, the official intellect of the Local Government Board, and the patience of the public. If the *City Press* may be relied upon, no less than 2,420 children have been attacked by this disease at Hanwell in less than eleven years. But if Mr. Holmes be right, and the history of ophthalmia at Hanwell dates back 25 years or more, who shall say how many children have suffered from the disease during that quarter of a century, and what is the amount of pain, danger, and injury they have been compelled to endure? Ophthalmia is a very painful, a very injurious, and a very contagious disease. Moreover, it is a disease that, though difficult to cure, is comparatively easy to prevent. Who, then, is to bear the blame of the Hanwell scandals, and what punishment should be meted out to the real offenders? It would be both instructive and important to know how many poor children have been disfigured for life as the result of their connection with Hanwell Schools, and how many have been rendered permanently and hopelessly blind. It is stated that there are now 1,200 children in the schools, and that about one-third (400) of them are in an ophthalmic condition. Not only so, but the same authority says that no fewer than 200 new cases occur annually—that is, four every school week. When it is remembered that patients afflicted with this disease suffer agonies on merely attempting to open their eyes to the light, and that the mildest treatment which can be adopted is so painful as to produce a severe shock to the patient's nervous system, and that, moreover, the treatment has sometimes to be continued for months in a darkened room, whilst education is for the time suspended, it is seen that the steady continuance of "ophthalmia at Hanwell" is a fact which demands some public attention. What is to be done? The immediate isolation of every case is urgently demanded, and must be insisted upon by a justly-incensed public opinion, if the local managers cannot find enough of mental energy within their own body to carry out so obvious and simple a plan. Not only so, but if it is found that the school precincts, after so long a prevalence of the disease, are so saturated with the poison that new cases continue to break out, the whole of the buildings should be razed to the ground or destroyed by fire. These may seem drastic remedies; but there is only one way of dealing with mad dogs, and that is to kill them. So, also, there is but one method possible with a building hopelessly poisoned with the germs of disease, and that is to destroy it utterly, with all that belongs to it.

**A Scandalous School History.**

Perhaps a man's value as a philanthropist may be measured by the degree in which he dispenses with mere sentimental garnitures in the public appeals he makes on behalf of his cause. Some hospital secretaries are past masters in the art of stale patheticality. Such are always to be looked upon with suspicion. The Rev. S. A. Barnett has attained to a distinguished eminence in the opposite art of extreme simplicity. If the perfection of art is to conceal art, the Vicar of St. Jude's, Whitechapel, is as nearly perfect in the statement of his "case for help" as a man may well be. Like the rest of us, he has been warmed and cheered—perhaps a little too much warmed in Whitechapel—by the brilliant, old-fashioned, "merrie month of May" we have just experienced. He feels as if he would like a sniff of the fresh breezes of country or sea. But he feels still more for the six hundred thousand children who crowd the various elementary schools of this vast London. He wants the public to help him to send away, for a brief country holiday, as many of the six hundred thousand as are stifled into feeble health and sickly pallor by bad air and poor food. He begins his very brief appeal by telling the public what was done last year. Seventeen thousand six hundred and thirty-seven London children had each a fortnight among the fields and woods during the summer. He says this country trip is to the child the "feature of the year's life" and "makes the whole year seem happier." We believe him. "Ten shillings is sufficient to give one child a holiday. Owners of clean and comfortable cottages in the country are, it appears, quite plentiful, who are willing to take in a little Londoner and feed and lodge and keep him clean for five shillings a week. Practical good Samaritans these cottagers are! "The plan of campaign," Mr. Barnett says, "is very simple. Visitors are appointed in every district to visit the schools, to select children whose only claim is their need, and to collect from the parents such payments as they can afford . . . Grants are then made from the general fund to the town visitors according to the needs of the district. By this plan the children go into the country feeling they are sent by friends, and not by a society, while a check is kept on those visitors who would keep for one district more than its fair share of good things. The children are anxiously looking for their holiday . . . All that is wanting is money." We do not need to say a single word on this appeal except one, and that is, that the treasurer is the Hon. A. Lyttelton, and that he is waiting, like the children, to receive subscriptions at 10, Buckingham-street, Strand, W.C.

**Ten Shillings a Child.**

Is it always true, as Gibbon said, that "persuasion is the resource of the feeble; but the feeble can seldom persuade"? If it be, then, alas, for the swallows and other helpless creatures on whom fashion has set her selfish and cruel hand! A man of science, who signed himself "Ornithologist," wrote a letter to the *Standard* the other day which would make every woman who ever wore bird or wing on her bonnet ashamed of herself if she had either a reasonable intellect or a gentle heart. It is not a sight which can be witnessed every day, that of a man of broad scientific knowledge and intelligence advancing with sober and pleading attitude to the ear of fashion to make appeal on behalf of defenceless birds. To denounce the senseless vulgarity which makes almost all women subordinate every kindly instinct and every whisper of reason and conscience to the overbearing authority of the prevailing mode, was probably his first thought. And well would he have been justified if he had given rein to that feeling. But his love of his feathered friends prevailed over his natural indignation, and apparently induced him to take that course which he thought more likely to serve their cause. He tells with affectionate minuteness the story of the confidence which the swallows have for thousands of years shown in protecting man. "They build their little mud mansions," he says, even in his very house if a window be left open. The present writer, however, knows of a place where they build in a little cottage every summer without the windows being left open. They go in and out by the door, and if it happens to be shut they will fly to it and peck at it with their beaks until it is opened for them. Their kind friend, Miss Raphael, who lives in the cottage in the summer months, never thinks it a trouble to let her little companions in and out. Whoever desires to see this sight for himself has only to pay a visit to The Lake, a little spot three miles from the town of Kirkcudbright, in the South of Scotland, and unless the birds have changed their habits since Midsummer, 1887, when the writer saw them, they will be found with their nest on the beam, from which it has not been removed for several years; and Miss Raphael will also be seen in the room as busy below as the swallows are above. But man, or rather woman, has broken the compact with the swallows which has existed for thousands of years, and the birds are beginning to be conscious of the fact, and to avoid those parts of the world where their destruction has been decreed. America is a shameful offender. A hundred and ten millions of birds are slaughtered there every year because fashion wills it. Half of these are worn by American women. In France the birds are butchered wholesale by electricity. In England our women follow the prevailing taste, and as many birds as are required are killed here, or imported from America or elsewhere. Those women who are so unfortunate as not to need to earn their own living seem to be growing steadily more inconsequent and unfeeling. They are like young cats, who must not only have their mouse, but must play with it for half an hour before finally killing and eating it. What man of any feeling will not prefer to choose his wife from among governesses, nurses, or other women who have been compelled to learn at least the elements of sense in the school of necessity?

